# A dataset of large ensemble of CMIP6-based transient climate scenarios for impact assessment in Great Britain

**DOI:** 10.1016/j.dib.2025.111695

**Published:** 2025-05-23

**Authors:** Mikhail A. Semenov, Nimai Senapati, Kevin Coleman, Adrian L. Collins

**Affiliations:** a Sustainable Soils and Crops, Rothamsted Research, West Common, Harpenden, AL5 2JQ, United Kingdom; b Net Zero and Resilient Farming, Rothamsted Research, West Common, Harpenden, AL5 2JQ, United Kingdom; c Net Zero and Resilient Farming, Rothamsted Research, North Wyke, Okehampton, Devon EX20 2SB, United Kingdom

**Keywords:** Climate change, Transient climate scenarios, CMIP6 ensemble, LARS-WG weather generator, Climate change impact assessment, Downscaling, SSPs

## Abstract

Under the extant threat of climate change, impact assessment studies are essential to investigate and quantify the severity of the potential impacts, and support recommendations for mitigation strategies with foresight. Future climate change scenarios are therefore crucial for underpinning impact studies. Here, transient climate scenarios are important as they provide a more realistic and dynamic evolution of future climate conditions over time, rather than only static climate scenarios. It is also important to downscale climate projection of Global Climate Models (GCMs) from coarse spatial and temporal resolution to local scale site-specific daily climate scenarios which have a sufficiently large number of years or realisations for accounting for inter-annual variability and detecting rare extreme climatic events. In the new dataset presented herein, transient future climate scenarios were generated at 26 representative sites across Great Britain (GB) using the Long Ashton Research Station Weather Generator (LARS-WG 8.0), based on climate projections from a subset of five GCMs from the latest Coupled Model Intercomparison Project Phase 6 (CMIP6) ensemble and two emission scenarios. For each site, 100 realisations of continuous transient time series of daily weather (minimum air temperature, maximum air temperature, rainfall and solar radiation) over the period 2020 to 2090 were generated. The use of a subset of five GCMs reduces computational requirements substantially for impact assessments, while allowing quantification of uncertainties in impacts related to uncertainty in future climate projections arising from GCMs. The dataset can be used to underpin assessments of future climate change risk and vulnerability, and their temporal patterns and progressive changes over time. Our data are designed to be used as a time series of climatic input to impact models for climate change assessments continuously over time related to various fields and disciplines, including land and water resources, agriculture and food production, soil carbon cycle, ecology and epidemiology, and human health and welfare. Various key stakeholders, such as researchers, breeders, farm managers, social and public sector advisers, policymakers and planners, may benefit from this new transient dataset for investigating, forecasting, designing and prioritising adaptive and mitigation strategies under changing climate.

Specifications Table

**Table:** 

Subject	Earth & Environmental Sciences
Specific subject area	Climate change; transient climate scenarios; downscaling of climate data from GCM and generating daily climate scenarios at local scale
Type of data	Table (.dat file and .st file) Raw model generated data (txt files)
Data collection	CMIP6 ensemble data: Future climate projections from the CMIP6 ensemble were obtained from the Copernicus Climate Data Store. https://cds.climate.copernicus.eu/cdsapp#!/dataset/projections-cmip6?tab=form Observed historical weather: Observed historical daily weather data at 26 representative sites across GB was obtained from the UK Met Office [1]. Baseline and transient future climate scenarios: One hundred yearly realisations of daily weather (minimum air temperature, maximum air temperature, rainfall and solar radiation) for baseline, and one hundred realisations of transient daily climate scenarios at the 26 selected sites were generated for the period 2020-2090 using a stochastic weather generator (LARS-WG 8.0) [2].
Data source location	Primary data sources: (i) Observed historical climate data: UK Met Office [1]. (ii) Future climate projections: CMIP6 ensemble, Copernicus Climate Data Store. https://cds.climate.copernicus.eu/cdsapp#!/dataset/projections-cmip6?tab=form
Data accessibility	The dataset [3] is available from - Repository name: Zenodo Data identification number: https://doi.org/10.5281/zenodo.14040993 Direct URL to data: https://zenodo.org/records/14040993
Related research article	None

## 1 Value of the Data

• The dataset [3] provides continuous transient future climate scenarios on daily time scale with sufficiently large numbers of realisations at local scale across GB, based on climate projections from a subset of five GCMs from the CMIP6 ensemble and two emission scenarios (SSP2-4.5 and SSP5-8.5). Each transient scenario for each site consists of 100 realisations of daily weather time series during the period 2020 to 2090. This large ensemble of CMIP6-based transient climate scenarios provides essential data, such as daily minimum and maximum air temperatures, rainfall and solar radiation, for climate change impact studies at local scale across GB for understanding the dynamic evolution of the impacts over time due to the time-dependent nature of these scenarios.
• The dataset could underpin assessments of future climate change risk, particularly their temporal patterns and progressive changes over time, for example risk and vulnerability of natural ecosystems, agroecosystems, water resources and infrastructure systems to heat waves, floods and droughts.
• The sufficiently large number of realisations in this new transient climate dataset could enable accounting of inter-annual variability and detection of rare extreme climatic events within various time scales (e.g., day, week, month, year, etc.) during 2020-2090.
• The use of a subset of GCMs reduces computational requirements substantially for impact assessments, while allowing explicit estimation of uncertainty propagation in impact assessments due to inherent uncertainties in GCMs.
• This new dataset can be used as a time series of climatic input to impact models for climate change assessments continuously over time in various fields, including land and water resources, agriculture and food production, soil carbon cycle, ecology and epidemiology, and human health and welfare. This dataset could help in explicit investigating and quantification of climate change legacy effects and non-linear feedback mechanisms over time.
• Various stakeholders, such as researchers, breeders, farm managers, social and public sector advisers, policymakers and planners, could benefit from this new transient climate dataset for designing and prioritising adaptive and mitigation strategies depending on the temporal patterns of impacts.

## 2 Background

Climate change is the single most critical issue of the 21st century [4]. Under ongoing climate change, impact studies are essential for assessing the severity of the potential impacts and supporting recommendations for mitigation strategies in advance. Future climate change scenarios are therefore crucial as an input for impact studies. Static future climate scenarios for a specific period, for example very near (e.g., 2030) or near future (2050), are important to estimate the climate change impacts at a particular point of time in details, for example climate scenarios as in Semenov et al. [5]. However, time-dependent climate change scenarios, i.e. climate time series or so-called transient climate scenarios, are critically important to investigate temporal patterns of the impacts, since they provide a more realistic and dynamic evolution of future climate conditions over time [6,7], rather than only static scenarios.

Climate change impact assessments often require a large ensemble of transient climate scenarios downscaled to local-scale, where each ensemble member represents plausible long weather series. A sufficiently large number of realisations is also needed for accounting for inter-annual variability and detecting rare extreme climatic events [5]. However, only very few realisations of transient climates are usually available for each GCM due to the high computational costs. Here, a potential viable solution can involve employing a stochastic weather generator [2] to downscale GCMs projections at local scale and generate a large ensemble of transient daily site-specific climate scenarios with a sufficiently large number of realisations based on a minimum workable subset of GCMs, covering the entire distribution and sensitivity of climate projections from GCMs ensemble. Such datasets are currently unavailable for GB. To address such a need, a large ensemble of the latest CMIP6-based [8] daily transient climate scenarios was generated for impact assessments in GB.

## 3 Data Description

**Fig 1:**
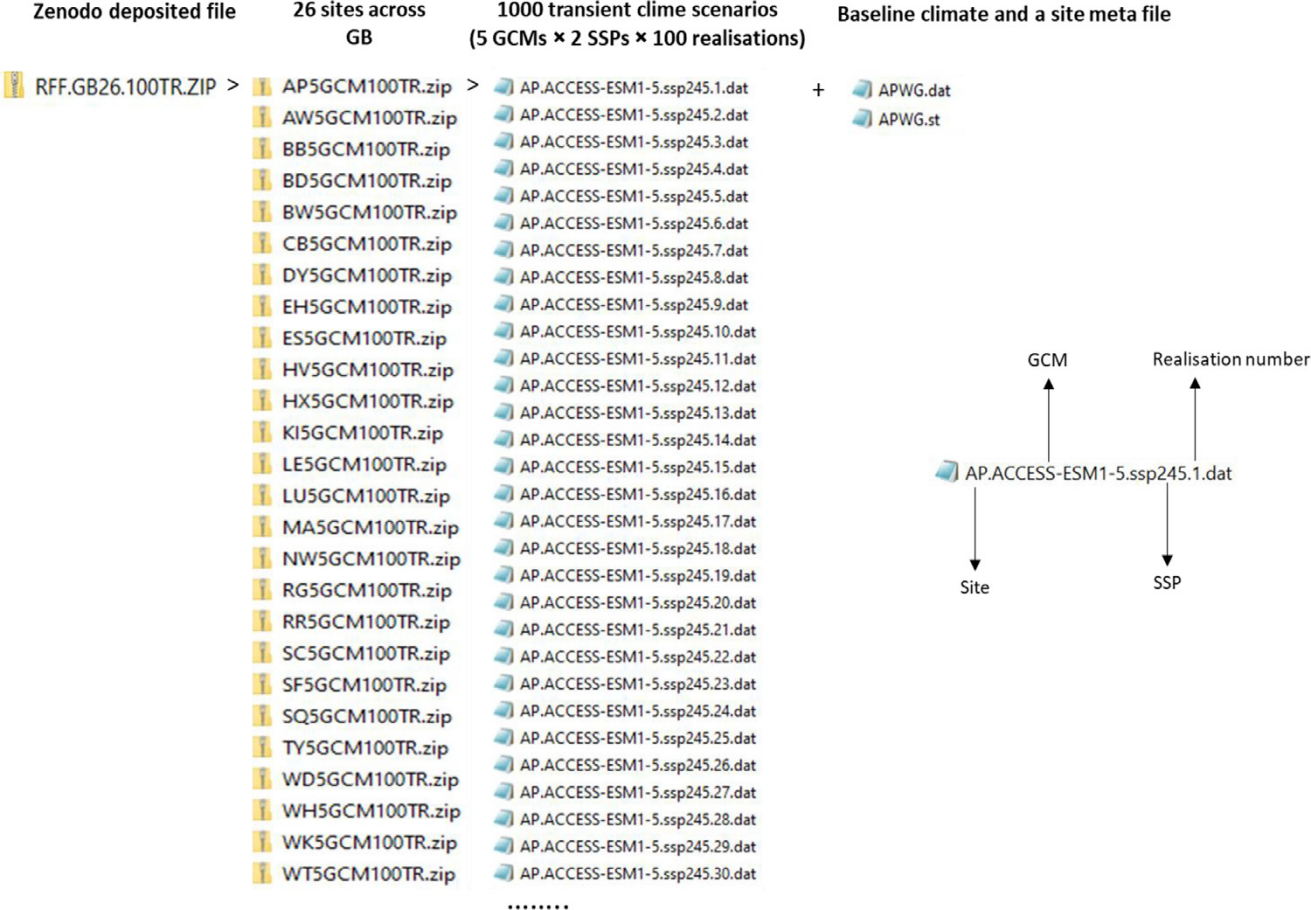
Description of the transient climate dataset [3].

**Fig 2:**
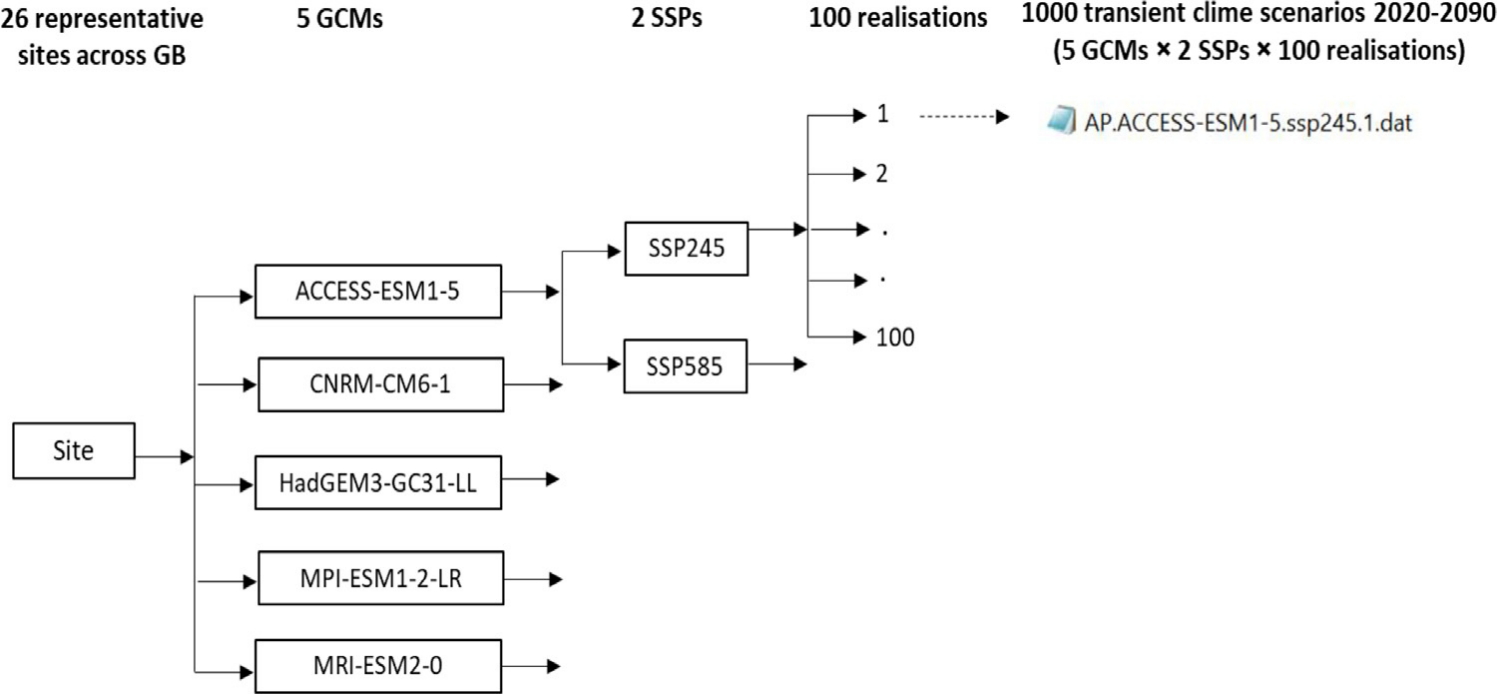
The structure of the transient climate scenarios spanning 2020 to 2090 at a representative site in the transient climate dataset [3].

**Fig. 3:**
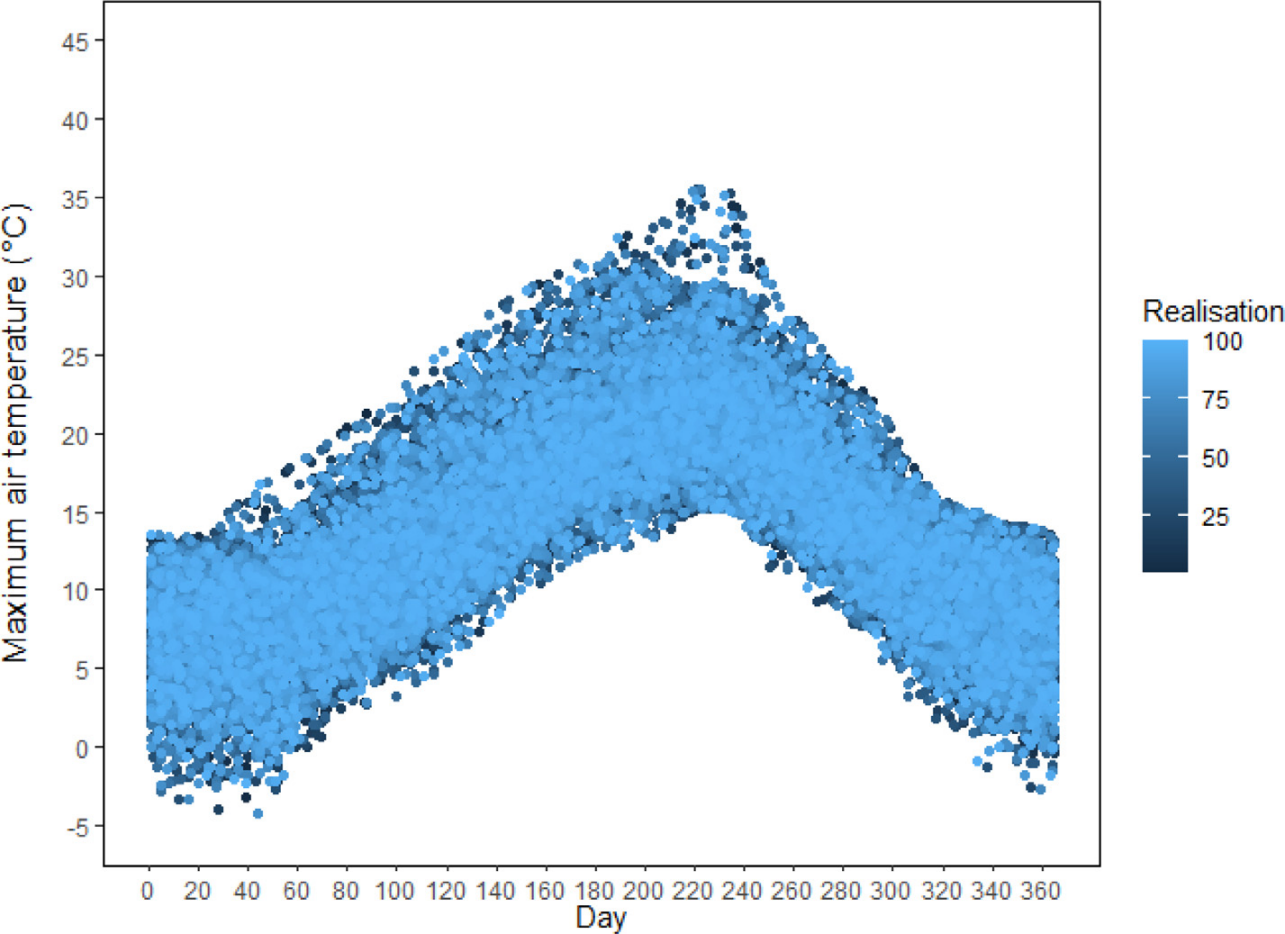
An example of a baseline climate at the Rothamsted (RR) site, representing 100 realisations of one climatic variable, i.e., daily maximum air temperature (°C), in a typical year representing the observed period 1985-2015 [data from the baseline climate file RRWG.dat inside the site folder RR5GCM100TR.zip].

**Fig. 4:**
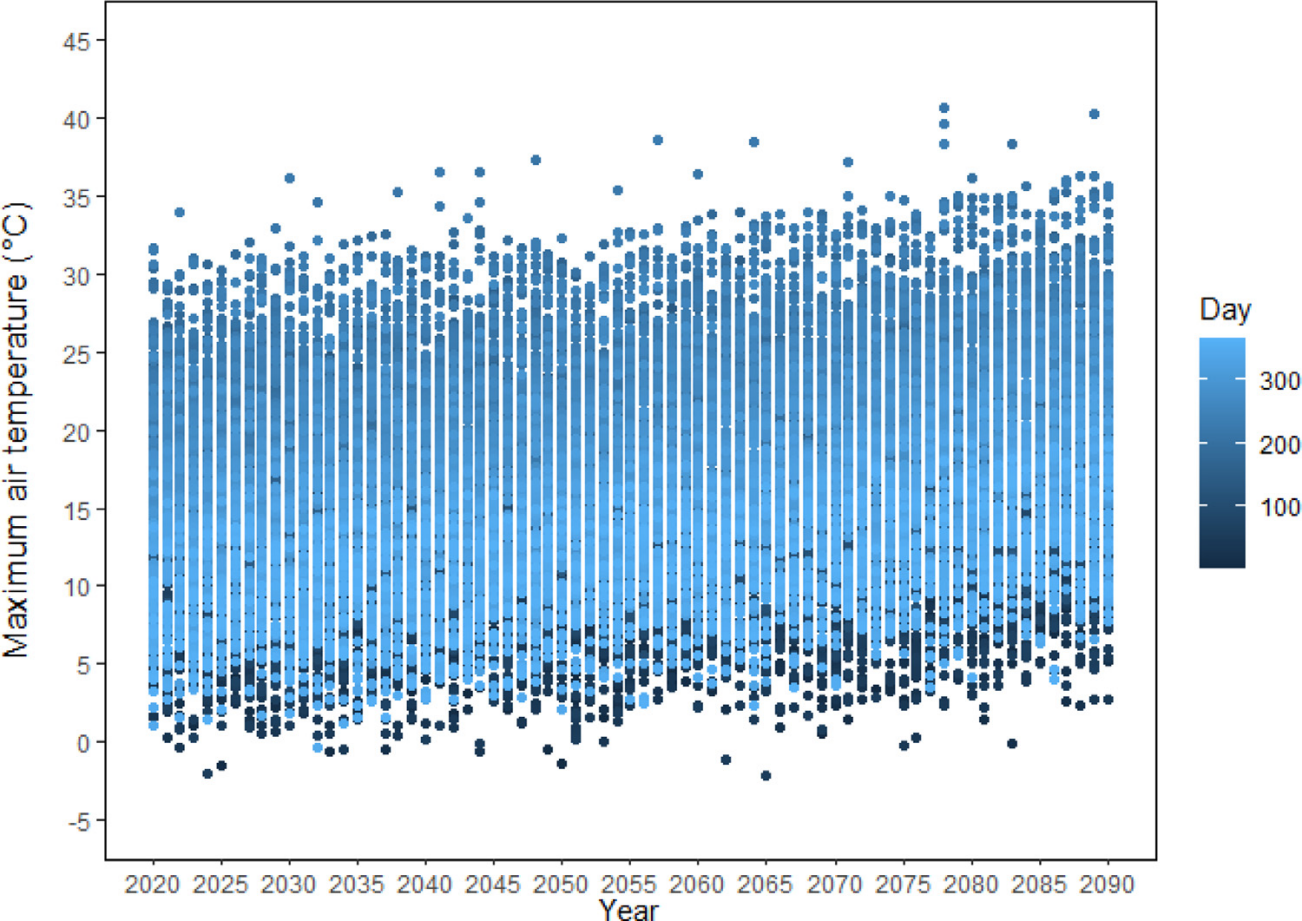
An example of a transient climate scenario at the Rothamsted (RR) site, representing the first realisation of one climatic variable, i.e., daily maximum air temperature (°C), during 2020 to 20290, based on one GCM (HadGEM3-GC31-LL) and one emission scenario (SSP5-8.5) [data from the climate scenario file RR.HadGEM3-GC31-LL.ssp585.1.dat inside the site folder RR5GCM100TR.zip].

This dataset [3] provides future continuous transient climate scenarios on daily time scale with sufficiently large numbers of realisations at local scale across GB, based on climate projections from the latest CMIP6 ensemble. The dataset consists of 100 realisations of continuous transient time series of daily weather over the period 2020 to 2090 at 26 representative sites across GB, based on a subset of five GCMs (ACCESS-ESM1-5, CNRM-CM6-1, HadGEM3-GC31-LL, MPI-ESM1-2-LR, and MRI-ESM2-0) selected from CMIP6 ensemble and two emission scenarios (SSP2-4.5 and SSP5-8.5). The dataset is deposited as a zip file “RFF.GB26.100TR.ZIP” in Zenodo, with notations indicating the name of the research project (Resilient Farming Futures: RFF), Great Britain (GB), 26 representative sites, 100 realisations and transient (TR) climate scenarios (Fig. 1). RFF.GB26.100TR.ZIP consists of 26 zipped folders for the 26 representative sites across GB. The file name for each site starts with the site abbreviation followed by “5GCM100TR”, indicating five selected GCMs and 100 realisations of transient climate scenarios (Fig. 1). Each site folder includes a total of 1002 files; viz., 1000 transient future climate scenarios over the time span 2020-2090 (5-GCMs × 2-SSPs × 100 realisations) [.dat files], one baseline climate [.dat file], and one site meta-file [.st] (Fig 1, Fig 2). The name of the future climate scenarios file (.dat) begins with the site abbreviation, followed by the name of the GCM, SSP, and the realisation number from 1 to 100. The name of the baseline climate file (.dat) consists of the site abbreviation followed by “WG”, meaning baseline generated by LARS-WG. The baseline climate at each site contains 100 realisations of daily weather for a typical year representing the observed period 1985-2015. The site meta file name starts with the site abbreviation followed by the abbreviation “WG”, meaning generated by LARS-WG. The site file contains information about the site (latitude, longitude and altitude), and climate scenarios (format, name and sequence of climatic variables). The baseline and each transient climate scenario contain four variables, viz., daily minimum air temperature, maximum air temperature, rainfall and solar radiation. The name, abbreviation, and units of the climatic variables in the dataset are described in Table 1. Fig. 3, Fig. 4 show an example of a baseline and transient climate scenario for a single weather variable (maximum air temperature) at the Rothamsted (RR) site.

**Table 1:** Climatic variables, abbreviations and units in the transient climate dataset.

Variable	Year	Day of year	Minimum air temperature	Maximum air temperature	Rainfall	Solar radiation
Abbreviation	YEAR	JDAY	MIN	MAX	RAIN	RAD
Unit	N/A	N/A	°C	°C	mm day^-1^	MJ m^-2^ day^-1^

## 4 Experimental Design, Materials and Methods

**Fig. 5:**
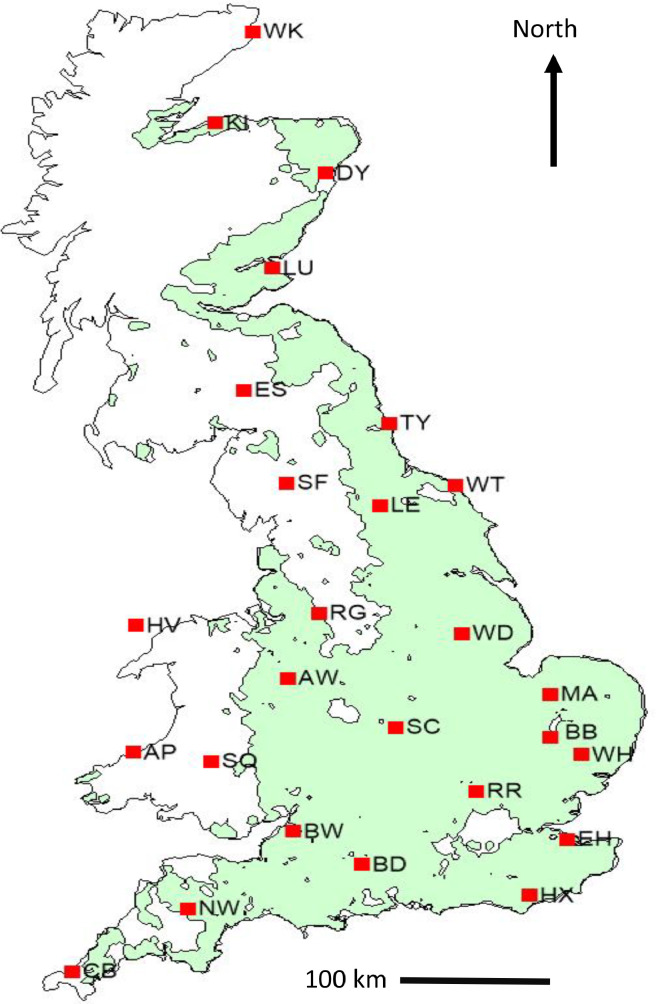
The selected representative sites in the transient climate dataset across Great Britain [3]. The green shading represents arable land. Please see details of the site information in Semenov et al. [5].

Sites selection across the GB. For this transient climate dataset [3], 26 representative sites across GB (Fig. 5) were selected from an available 85 climate stations within the Met Office network [1]. These sites were selected based on: (1) the missing data in obeservations should be < 10 % for temperature and precipitation, and (2) sites should be representative of diverse locations and climates across GB, providing broad and even coverage of its arable land [5]. According to the Köppen-Geiger climate classification for GB, climate predominantly falls into the Cfb climate type, which is a warm temperate climate with a maritime influence. This indicates GB experiences relatively mild winters and cool summers with no significant dry season and at least 75 % of the precipitation occurring evenly throughout the year. While Cfb is the dominant climate, some areas, particularly in the south and southeast, might see shifts towards more Mediterranean-like climates (Csa or Cs) due to increasing temperatures.

**Baseline climate.** The daily observed weather during the period 1985–2015 are available at each selected site [1,5]. To generate a baseline at each site, observed weather data were used to estimate site-specific climatic parameters required by a stochastic weather generator (LARS-WG 8.0) [2]. Adequate reproduction of climatic variability and change by LARS-WG, including extreme climatic events at local scale, have been reported by various previous studies [5,[9], [10], [11], [12]]. To generate a reasonably large number of years of the baseline, for accounting for inter-annual variability and detecting climatic extreme events, 100 years of daily weather representing typical years for the period 1985–2015 at each site were generated using LARS-WG, based on estimated site-specific parameters; hereafter, defined as the ‘baseline climate’. The baseline climate has statistical characteristics similar to the observed weather at each site, with probability distributions close to those of the observed climate. LARS-WG automatically computes various statistics, including KS-test statistics, t-statistics and f-statistics, and their corresponding p-values which allows to compare observed and generated weather [2].

**Future transient climate scenarios based on CMIP6 ensemble.** Future transient climate scenarios were based on five GCMs from the latest CMIP6 ensemble [8]. A total of 15 GCMs from the CMIP6 ensemble were integrated into LARS-WG 8.0 [2]. Based on their performance over northern Europe including the UK, climate sensitivity and the distribution of GCMs, a subset of the best five performing GCMs (Table 2) was selected for the present dataset [5,13]. The selected five GCMs are evenly distributed among the 15 GCMs, capturing uncertainty in climate projections from the CMIP6 ensemble [5]. The use of a subset of five GCMs substantially reduces computational time for impact assessment studies, while allowing explicit estimation of uncertainties in impacts related to uncertainty in future climate projections due to the GCMs. Two future emission scenarios, defined as Shared Socioeconomic Pathways (SSPs), were selected for this transient climate dataset to cover the range of possible future development of anthropogenic drivers of climate change; viz., (i) an intermediate GHG emission scenario: SSP2-4.5 – Middle of the Road (medium challenges to mitigation and adaptation), with an additional radiative forcing of 4.5 W m^-^² by the year 2100, and (ii) a very high GHG emission scenario: SSP5-8.5 – Fossil-fuelled Development – Taking the Highway (high challenges to mitigation, low challenges to adaptation), with an additional radiative forcing of 8.5 W m^-^² by the year 2100 [14]. LARS-WG downscales climate projections from the GCMs and incorporates changes at local scale in the mean climate, climatic variability and extreme events derived from the GCMs by modifying the statistical distributions of the weather variables at each site with delta-changes in climatic variables derived from the GCMs [2,10,15]. The monthly outputs from GCMs were used to calculate delta-changes which were used to perturb site-specific parameter distributions generated by LARS-WG. For each site, GCM and SSP, 100 realisations of transient daily weather for the period 2020-2090 were generated using LARS-WG 8.0, based on climate projections from the five selected GCMs.

**Table 2:** The five Global Climate Models (GCMs) from the Coupled Model Intercomparison Project Phase 6 (CMIP6) multi-model ensemble used in the construction of the transient climate scenarios for Great Britain.

GCM	Country of origin	Research centre	Grid resolution: latitude x longitude	Reference
ACCESS-ESM1-5	Australia	Commonwealth Scientific and Industrial Research Organisation (CSIRO)	1.25° x 1.875°	[16]
CNRM-CM6-1	France	Centre National de Recherches Meteorologiques (CNRM), Centre Europeen de Recherche et de Formation Avancee en Calcul Scientifique (CERFACS)	1.40° x 1.406°	[17]
HadGEM3-GC31-LL	UK	UK Met Office Hadley Centre (MOHC)	1.25° x 1.88°	[18]
MPI-ESM1-2-LR	Germany	Max Planck Institute for Meteorology (MPI-M)	1.39° x 1.41°	[19]
MRI-ESM2-0	Japan	Meteorological Research Institute (MRI)	1.113° x 1.125°	[20]

## Limitations

The potential uncertainty in transient climate scenarios due to inherent uncertainties in GCMs was captured by using an ensemble of 5 GCMs. However, only one weather generator, LARS-WG, was used. It might be beneficial to quantify uncertainty related to the use of different weather generators for construction of transient climate scenarios. This will allow to estimate overall uncertainty from both sources in future transient climate scenarios for impact assessment.

## Ethics Statement

The authors have read and follow the ethical requirements for publication in Data in Brief and confirming that the current work does not involve human subjects, animal experiments, or any data collected from social media platforms.

## CRediT Author Statement

**Mikhail A. Semenov:** Conceptualization, Methodology, Software, Validation and Data Curation. **Nimai Senapati:** Conceptualization, Writing - original draft, Writing - review & editing. **Kevin Coleman:** Conceptualization, Writing – review & editing. **Adrian L. Collins:** Conceptualization, Writing – review & editing.

## Data Availability

ZenodoA large ensemble of CMIP6-based transient climate scenarios for impact assessment in Great Britain. (Original data).

## Appendix Supplementary materials

Image, application 1

## References

[1] Met Office, The UK climate - Synoptic and climate stations. UK. 2023. https://www.metoffice.gov.uk/research/climate/maps-and-data/uk-synoptic-and-climate-stations

[2] Semenov M.A. (2024).

[3] Semenov M.A., Senapati N., Collins A.L. (2024).

[4] Lee H., Romero J., IPCC, Core Writing Team (2023). Climate Change 2023: synthesis Report. Contribution of Working Groups I, II and III to the Sixth Assessment Report of the Intergovernmental Panel on Climate Change.

[5] Semenov M.A., Senapati N., Coleman K., Collins A.L. (2024). A dataset of CMIP6-based climate scenarios for climate change impact assessment in Great Britain. Data Brief.

[6] Meyer A.S. (2024). Temporal dynamics of climate change exposure and opportunities for global marine biodiversity. Nat. Commun..

[7] Mudelsee M. (2019). Trend analysis of climate time series: a review of methods. Earth-Sci. Rev..

[8] Eyring V. (2016). Overview of the coupled model intercomparison project phase 6 (CMIP6) experimental design and organization. Geosci. Model Dev..

[9] Gitau M.W., Mehan S., Guo T. (2018). Weather generator effectiveness in capturing climate extremes. Env. Process.

[10] Semenov M.A. (2010). ELPIS: a dataset of local-scale daily climate scenarios for Europe. Clim. Res..

[11] Semenov M.A. (2008). Simulation of extreme weather events by a stochastic weather generator. Clim. Res..

[12] Semenov M.A., Brooks R.J., Barrow E.M., Richardson C.W. (1998). Comparison of the WGEN and LARS-WG stochastic weather generators for diverse climates. Clim. Res..

[13] Bradshaw C., Cottrell A., Crocker T., Falloon P. (2023).

[14] Riahi K. (2017). The shared socioeconomic pathways and their energy, land use, and greenhouse gas emissions implications: an overview. Glob. Env. Change.

[15] Semenov M.A., Barrow E.M. (1997). Use of a stochastic weather generator in the development of climate change scenarios. Clim. Change.

[16] Ziehn T. (2019).

[17] Voldoire A. (2018).

[18] Roberts M. (2017).

[19] Wieners K.-H. (2019).

[20] Yukimoto S. (2019).

